# Poor Sleep Quality, Depression and Social Support Are Determinants of Serum Phosphate Level among Hemodialysis Patients in Malaysia

**DOI:** 10.3390/ijerph17145144

**Published:** 2020-07-16

**Authors:** Eileen Suk Ying Ng, Poh Yoong Wong, Ahmad Teguh Hakiki Kamaruddin, Christopher Thiam Seong Lim, Yoke Mun Chan

**Affiliations:** 1 Department of Nutrition and Dietetics, Faculty of Medicine and Health Sciences, Universiti Putra Malaysia (UPM), Serdang 43400, Malaysia; eileen_ensy07@hotmail.com (E.S.Y.N.); wpohyoong@gmail.com (P.Y.W.); drhakiki194@gmail.com (A.T.H.K.); 2 Department of Medicine, Faculty of Medicine and Health Sciences, Universiti Putra Malaysia (UPM), Serdang 43400, Malaysia; christopher@upm.edu.my; 3 Research Center of Excellence, Nutrition and Non Communicable Diseases, Faculty of Medicine and Health Sciences, Universiti Putra Malaysia (UPM), Serdang 43400, Malaysia; 4 Malaysian Research Institute on Ageing, Universiti Putra Malaysia, Serdang 43400, Malaysia

**Keywords:** hyperphosphatemia, sleep duration, expression, social support, hemodialysis patients

## Abstract

Despite optimal control of serum phosphate level being imperative to avoid undesirable health outcomes, hyperphosphataemia is a highly prevalent mineral abnormality among the dialysis population. This study aimed to determine factors associated with hyperphosphatemia among hemodialysis patients in Malaysia. Multiple linear regression analysis was used to ascertain the possible factors that influence serum phosphate levels. A total of 217 hemodialysis patients were recruited. Hyperphosphatemia was prevalent. Only approximately 25% of the patients were aware that optimal control of hyperphosphatemia requires the combined effort of phosphate binder medication therapy, dietary restriction, and dialysis prescription. The presence of diabetes mellitus may affect serum phosphate levels, complicating dietary phosphorus management. Patients who were less depressive portrayed higher serum phosphate levels, implying intentional non-compliance. Better compliance on phosphate binder, longer sleep duration, and higher social support was associated with a lower level of serum phosphate. Despite sleep disturbance being one of the most prevalent and intense symptom burdens identified by hemodialysis patients, relatively few studies have addressed this issue. It is time to formulate sleep therapeutic interventions besides the encouragement of strong social support, hoping which many clinical outcomes including hyperphosphatemia can be better controlled among hemodialysis patients.

## 1. Introduction

Kidney failure is a worldwide public health problem, with increasing incidence and prevalence, high health care costs, and poor outcomes [1,2,3]. Progressive deterioration of kidney function among patients with end-stage renal disease (ESRD) results in elevated serum phosphorus concentrations which have been associated with a number of clinical complications including abnormal bone and mineral metabolism, soft tissue and vascular calcification, cardiovascular morbidity and mortality [4], with a significant impact on health care costs [5].

Patients on hemodialysis (HD), the most common treatment modality, are required to adhere strictly to treatment regimen namely diet, fluids, medications (phosphate binder), and dialysis therapy [6]. The most common dialysis prescriptions, however, do not provide adequate phosphate removal [7], with hyperphosphataemia frequently reported among HD patients [8,9,10]. Precise reasons for this gap remain unclear and various factors have been linked to poor phosphate compliance among HD patients including socio-demographic factors [11,12], depression [13,14], social support [15,16], medication adherence [11,17,18], nutritional knowledge on phosphorus [11,19], and sleep quality [20,21] with inconsistencies existing [22,23,24]. A burgeoning body of research has greatly advanced our understanding of the manifestations and management of kidney diseases, nevertheless, hyperphosphatemia remains prevalent among HD patients in Malaysia [11,25,26]. Despite hyperphosphatemia being prevalent, there is a paucity of data pertaining to potential determinants of hyperphosphatemia among HD patients in the local setting. The current practice of phosphate management is largely emphasized on the use of phosphate binders, with the dietary management of phosphorus and dialysis as adjuncts [27]. In view of the notably low adherence of phosphate binders and the complexity of dietary management, sub-optimal control in phosphate management among HD patients is expected. Inevitably, patients’ factors such as their perceived social support and depressive symptoms have been implicated as influential drivers of compliance behavior [27], but this has not been addressed adequately in the routine phosphate management of patients. Taken together, the present study aimed to determine the factors influencing serum phosphate among HD patients, hoping the research outcomes can optimize the existing management of serum phosphate among dialysis patients. 

## 2. Materials and Methods

This study was conducted among HD patients receiving treatment at dialysis centers. Hemodialysis involves diverting blood into an external machine, made up of a series of membranes that act as filters. During hemodialysis, the membranes will filter waste products such as phosphate, potassium, and urea. During this process, important nutrients such as amino acids will be removed as well, resulting in a high prevalence of malnutrition among hemodialysis patients. Filtered blood is passed back into the patient’s body. Most patients require dialysis three times a week on alternate days, with each session lasting for four hours. Data collection was performed while patients were on dialysis as most patients would prefer to rush home after dialysis. During dialysis sessions, patients can sit or lie on a couch, recliner, or bed depending on the setting of the dialysis centers. Some patients may feel a bit sick, dizzy, and may have muscle cramps during the procedure, hence, data collection was performed only when patients were comfortable and at ease. 

### 2.1. Subjects

**Figure 1:**
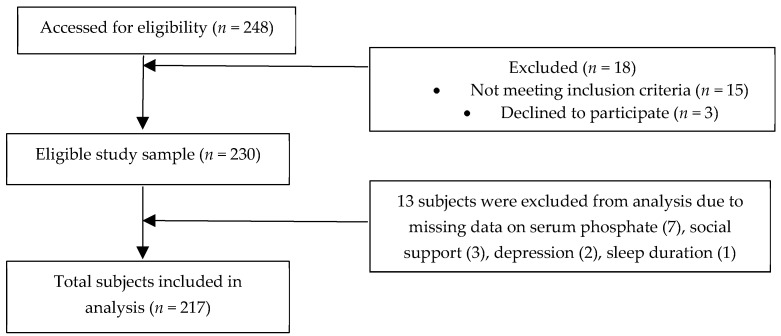
Consort Flow Diagram.

The present study is a cross-sectional study whereby 230 eligible patients were recruited from seven selected HD centers via multistage sampling. The inclusion criteria entailed all Malaysians HD patients aged 18 years and above, able to understand and speak Mandarin, English or Malay languages and had undergone HD treatment for at least 3 months. Non-Malaysians and HD patients with dementia and mental illness, acute chronic or chronic hepatitis B and C, history of parathyroidectomy as well as recent hospitalization due to complications related to hemodialysis (e.g., vascular access-related infection or bleeding, hypotension or hypertension, pulmonary edema, and or cardiac arrhythmias) were excluded from this study. There were 13 patients with incomplete data and were excluded from the analysis. This study was performed in accordance with the principles of the Declaration of Helsinki. Ethical approval for the study protocol was obtained from the university’s Ethics Committee for Research Involving Human Subjects (JKEUPM-2018-231) while approvals were gained from the respective HD centers. Eligible patients were provided with a subject information sheet and written informed consent was obtained from all eligible patients prior to data collection with the assurance of confidentiality and anonymity. Figure 1 depicts the consort diagram of the study. 

### 2.2. Instrumentation

A pre-tested structured questionnaire was used to ascertain information on socio-demographic characteristics and clinical background of patients. Beck Depression Inventory (BDI) [28] was used to assess the severity of depressive symptoms among the patients. A universal recognized reliable tool for assessment of depression among hemodialysis patients, revealing 91% sensitivity and 86% specificity [29], BDI consists of 21 items with the first 13 questions concerning the cognitive-affective area, while other questions concern the somatic problems accompanying mood disorders. Each question is measured via a 4-point scale (0–3), which gives a total possible score of 0 to 63. Patients were classified into four levels of depression, namely none or minimal depression, mild to moderate depression, moderate to severe depression, or severe depression. 

Perceived social support of patients from three aspects (family, friends, and significant others) was assessed by using the 12-item Multidimensional Scale of Perceived Social Support (MSPSS) [30]. Response for each item was scored on a 7-point Likert scale (very strongly disagree to very strongly agree). The MSPSS was divided into 3 subscales, which were Family Subscale, Friends Subscale, and Significant Others Scale along with a Total Score Scale. The total possible score ranges from 12 to 84, with a higher score indicating greater social support perceived by an individual. The level of perceived social support for each subscale and the total score scale was classified into low, moderate, or high, accordingly. 

Medication adherent (specifically referred to phosphate binder) was ascertained as missing any dosage of phosphate binders for the past one week. The 6-item Simplified Medication Adherence Questionnaire (SMAQ) [31] was adopted and adapted to further ascertain common barriers leading to non-adherence of phosphate binder. Nutritional knowledge on phosphorus (renal diet and phosphorus control) was adapted from Karavetian, Abboud, Elzein, Haydar, and Vries [32]. Questions assessing the nutritional knowledge of patients regarding phosphorus control and renal diet were adapted in the study. Multiple responses were allowed for selected questions. The total score ranged from 0–11 and was converted into a percentage and a cut-off score of 60% indicated sufficient knowledge. One score was given to each question answered correctly with all the correct answer options being selected. 

Sleep quality of patients in the past month was ascertained using the validated Pittsburgh Sleep Quality Index (PSQI) [33]. The PSQI is composed of seven components, namely subjective sleep quality, sleep latency, sleep duration, sleep efficiency, sleep disturbances, sleep medication use, and daytime dysfunction. Each sleep component has a scale factor from 0 to 3, and the seven components collectively form a global score ranging from 0 to 21. A higher global PSQI score indicates lower sleep quality. A global PSQI score of ≤5 indicates satisfactory sleep quality, whereas a score of >6 indicates poor sleep quality. Cronbach’s alpha of this index was 0.823, indicating that the instrument has acceptable internal consistency. 

Objective adherence to phosphate was obtained retrospectively from the medical records using the pre-dialysis serum phosphate levels. Patients were considered non-adherent when the average of the pre-dialysis serum phosphate levels (past nine months from the day of data collection) exceeded 1.6 mmol/L based on recommendations from Clinical Practice Guidelines on Renal Replacement Therapy [34]. Other key clinical parameters pertaining to HD such as serum calcium, parathyroid hormone, alkaline phosphatase, duration of dialysis, and adequacy of dialysis (Kt/V) were ascertained in the study. 

### 2.3. Data Analysis 

All data analyses were performed using SPSS Version 24.0 (IBM Corp., Armonk, NY, USA) [35]. Descriptive statistics were used to present socio-demographic characteristics, clinical factors, depression level, sleep duration, social support, nutrition knowledge on phosphorus, and serum phosphate level. Variables with *p* < 0.25 in the simple linear regression [36] were entered in the stepwise multivariate model to determine the contribution of socio-demographic characteristics (age, employment, income), duration of sleep, presence of comorbidities, medication adherence, depression, and social support to serum phosphate level. All tests were two-sided, with a significance level set at 0.05. 

## 3. Results 

A total of 217 eligible patients were recruited with their characteristics depicted in Table 1. The mean age of the patients was 57 years old, made up of a comparative proportion of men and women. The majority of our patients were married (78.3%) and possessed a secondary education level (47.9%). Most of them were retirees (47.0%) and approximately 91.0% of the patients had low and middle household incomes. Hypertension was most prevalent (81.1%) followed by diabetes mellitus (55.8%). Mean HD vintage of patients was approximately 11 years. 

With regards to depression, the mean BDI score attained by the patients was 5.76 ± 5.66 with approximately one-quarter of them presented with mild to moderate and moderate to severe depression, respectively. In addition, there were approximately three-quarters of the patients perceived a high level of social support with merely 2% of the patients perceived themselves as having a low level of social support. On the other hand, a majority of the patients perceived a high level of social support on Family (87.6%) and Significant Others (80.6%) subscales but perceived relatively less support from Friends (58.5%). The mean duration of sleep of the patients was approximately 5.6 h per day, with less than half of the patients slept more than 6 h a day, while another one-third of them slept less than 5 h per day. This could be a worrying scenario as the majority of the subjects did not achieve the recommended sleep duration of 7 h for optimal health as proposed by the American Academy of Sleep Medicine. 

Means serum calcium, phosphate, iPTH, and Kt/V were comparable with national renal registry data. Serum calcium was normal in 56% of the subjects, followed by approximately a quarter of the patients with hypocalcemia and hypercalcemia, respectively. The mean serum phosphate level of patients was 1.83 ± 0.50 mmol/L, with 77.4% of them failing to achieve the recommended target range of serum phosphate. There was 84.3%, 72.8%, 53.5%, and 27.8% of the patients’ perceived optimal serum phosphate control depending on a low phosphorus diet, phosphate binder, and dialysis process, and all three aspects respectively. All the patients were on a calcium-based phosphate binder. Approximately 70% of the patients had difficulties to adhere to phosphate binders in the present study, with more than half to approximately two-thirds of them forgetful and careless when taking phosphate binder medications. There were another 40% who had issues with the high pill burden and adverse effects of the phosphate binder (gastrointestinal discomfort such as constipation and unpleasant taste). A total of 94.0% of the patients scored less than 60% on nutrition knowledge on phosphorus, reflecting insufficient knowledge on phosphorus. This was affirmed despite the majority of patients (84.3%) acknowledging the importance of avoiding phosphate-rich foods in serum phosphate controls, while only approximately 13% were able to identify foods with high phosphorus content (data not shown). 

Table 2 shows the single and multiple linear regression (stepwise method) analysis of sociodemographic factors (age, employment status, household income), clinical and medical factors (adequacy of dialysis, presence of co-morbidities, adherence on phosphate binder, duration of dialysis), sleep duration, depression, social support, nutritional knowledge on phosphorus and serum phosphate levels. Only variables that resulted in a p-value of less than 0.25 at the simple linear regression were selected for the multiple linear analysis. Adequate dialysis (β = −0.413, *p <* 0.05), adherence to phosphate binder (β = −0.368, *p <* 0.05), age (β = −0.305, *p <* 0.05), sleep duration (β = −0.288, *p <* 0.05), presence of diabetes mellitus (β = 0.272, *p <* 0.05), presence of depression (β = −0.265, *p <* 0.05), and social support (β = −0.141, *p <* 0.05)significantly contributed to serum phosphate level among hemodialysis patients after controlling for sex, as serum phosphate level was significantly different between male and their female counterparts. The prediction model was statistically significant (F = 12.4, *p* < 0.05) with the factors above accounting for 23.6% of the variance in serum phosphate level. There were no significant correlations between nutrition knowledge on phosphorus with serum phosphate levels.

Table 3 depicts serum phosphate of patients according to specific variables which contributed significantly to serum phosphate in the multiple linear regression. Patients who adhered to phosphate binder prescription, older persons (defined as more than 60 years of age), diabetics, patients with adequate dialysis, depression, and higher social support had lower serum phosphate than their counterparts. While patients who slept less than 5 h per day had significantly higher serum phosphate than their counterparts, there was comparable serum phosphate between patients who slept 6 h per day and those sleeping equal to or more than 7 h. 

## 4. Discussion 

Hyperphosphataemia is a secondary complication in established renal failure patients. Approximately 80% of the patients in this study had elevated serum phosphate levels, which echoed earlier studies and reaffirmed the challenge in optimal control of serum phosphate levels. The unemployment rate of patients in this study was more than 76%, which is unexceptionally high considering only approximately 40% of the patients were aged more than 60 years old, a retirement age for a Malaysian. This is a universal scenario as HD patients often require an early retirement or sacrifice their employment or job opportunities to fit in the HD schedule [37,38]. Loss of productivity due to unemployment among HD patients had been reported to be higher compared to peritoneal dialysis [39,40], besides reduced quality of life attributed to the necessity for frequent travel.

Approximately one-quarter of the patients had depression, which was congruent with the reported prevalence rate of depression of 20% to 30% [29,41], despite being lower than the previous local study [42]. However, it should be noted that a different diagnostic tool was used in the previous study, making direct comparison not possible. Findings in our present study dictated most of the patients had none or minimal depression, which was contradicted with others [43,44,45,46] who found that most hemodialysis patients exhibited mild to moderate depression. The plausible reason to this discrepancy could be due to the overlap between symptoms of depression and uremic symptoms related to end-stage renal failure, resulting in patients that presented with uremic syndrome may be screened positive for depression, especially when self-reported measures were being utilized [47,48,49]. This present study utilized an interviewer-administered questionnaire method, which permitted the clarification of the differences to the patients. For social support, the high level of social support level perceived by the subjects were comparable to other studies [50,51]. Culture and religious beliefs that emphasized the role of family during times of sickness may contribute to the high level of family support attained [52,53]. The high level of perceived social support by patients may partly explain the low level of depression among this cohort.

Optimization of the phosphate binder used by patients with ESRD to achieve target serum phosphorus levels toward the recommended range is of utmost importance to minimize morbidity and mortality risks [54]. Nevertheless, nonadherence to phosphate binder is common with an estimation of up to 74% of ESRD patients are noncompliant to phosphate binder medication therapy [55]. Forgetfulness and carelessness were the main contributors to unintentional medication adherence among patients in this study, which was congruent with previous studies [11,56,57,58]. Adverse effects associated with phosphate binders such as gastrointestinal discomfort and high pill burden [11,59], necessity for strict adherence to the timing and dosing of the phosphate binder medication along with a busy social life and schedule may further aggravate this condition [58,60]. Considering polypharmacy is a common phenomenon among HD patients [61,62] due to the presence of various complications and underlying issues of the patients which may attribute to poor medication adherence, future studies focusing particularly on optimal clinical targets and lenient treatment strategies are warranted. On the other hand, a shared or active decision-making approach has been shown to improve patients’ medication adherence [63] and such an approach should be considered for hemodialysis patients who require long term continuum of care. Use of electronic monitoring devices to remind patients to take their medications at prescribed times may be helpful in improving medication adherence among forgetful patients [64].

Theoretically, patients with better knowledge possess higher awareness and are able to make better choices compared to lower knowledge patients. Several studies also have highlighted the lack of knowledge as a contributory factor to poor phosphate control, despite the association between phosphorus nutrition knowledge and serum phosphorus level being equivocal [65]. Similar to our findings, previous studies have reported better knowledge does not always translate to a better serum phosphate level [11,22,65,66,67]. With the growing body of evidence that educating hemodialysis patients on phosphorus improved serum phosphate levels [68,69,70,71], it is incumbent upon the healthcare professional community to acknowledge that education programs are indispensable for the optimal management of hemodialysis patients in routine care [72]. On the other hand, it would be appropriate to consider the application of more interactive approaches or technologies such as the internet and mobile telephones as education tools to extend support beyond the hospital setting, and enhancing accessibility to information aims to improve patients’ self-care management [66]. The lacking of association between better knowledge and better serum phosphorus control further suggests the use of educational empowerment techniques such as cognitive behavior interventions [73] or patient-centred approaches such as motivational interviewing [74] may be superior to the traditional approaches of information giving. Prevalence of poor nutritional knowledge on phosphorus among the patients was extremely high and worrying, which was in agreement with previous studies [11,19,22,67,75,76]. Complexity of the dietary regimen as well as the rigidity and lack of freedom that reduces the enjoyment and pleasure while dining might contribute to the failure to achieve the recommended target range of serum phosphate of the patients [22,57,77]. Emerging user-friendly educational initiatives such as motivational interviewing techniques and “traffic light” scheme to classify foods based on low, intermediate, or high phosphorus content may be useful. Empowerment of patients to tailor the phosphorus content of food to their phosphate binder use per meal will help in achieving optimal control of hyperphosphatemia while reducing the need for stringent dietary restrictions [78].

A younger age was associated with higher serum phosphate levels which was congruent with previous studies [79,80,81,82]. A difference in the lifestyle between older and younger patients along with self-perception of being less vulnerable to complications of non-compliance might have contributed to this [6,82,83,84]. Increased undernutrition with age, decreased renal phosphate reabsorption, and hormonal factors may also attribute to this [85]. Duration of dialysis was not associated with serum phosphate in the present study which was contradicting with evidence before this [11,86]. Earlier studies showed that as time passes, ESRD patients may easily get frustrated with the need to comply with long lists of restrictions [87], resulting in higher serum phosphate. We do not have an exact explanation but it is postulated that the heterogeneity of the patients with a wide range of duration of dialysis may have attributed to this discrepancy. 

While it is well known that ESRD prevalence is increasing with the rise in the number of diabetic nephropathy patients, presence of diabetes mellitus was significantly correlated with serum phosphate levels in the present study. This finding was incongruent with previous studies [88,89] with inconsistencies existed [85,90]. Earlier studies revealed that medicines prescribed for the treatment of diabetes mellitus may incorporate highly bioavailable inorganic phosphate as an additive, which attributes to the elevation of serum phosphate levels [91]. Nevertheless, the exact mechanism concerning hyperphosphataemic episodes in diabetic hemodialysis patients remains unclear and requires more extensive and in-depth studies in the future. More studies are warranted to evaluate the precise association between diabetes and phosphatemia and its mechanism [85].

Evidences are growing that hyperphosphatemia was associated with poor sleep quality [20,21,92,93], despite inconsistencies existing [24]. While the presence of hyperphosphatemia-related pruritus could be the mediating factor for poor sleep quality [20], to the best of our knowledge, there is no clear mechanism of how poor sleep quality may influence serum phosphate level among the dialysis population. In light of the challenges of optimal control of hyperphosphatemia, despite the use of a new generation of phosphate binders and dialysis membranes, the findings of this study signify more work is needed on how sleep interventions may affect serum phosphate level as well as the possible mechanisms. 

Our finding shows that less depressive hemodialysis patients may have a higher serum phosphate level, which was consistent with earlier studies [9,94,95,96,97]. It is possible that patients who were more carefree and positive may perceive themselves as less vulnerable to the risks of non-compliance, hence exercising more freedom in their diet or medication, resulting in higher serum phosphate levels. In light of the presence of psychiatric disorders such as depression, which have often been associated with a higher likelihood of adverse clinical outcomes including hospitalization and mortality in CKD patients, our findings should be interpreted carefully and more studies are needed to delineate the potential associations between depression and clinical outcomes including serum phosphate level. Besides, subjects in our study might perceive themselves as having stronger self-willpower in controlling their serum phosphate level, hence social support did not seem to exert a great impact on their phosphate compliance. Our finding was in accordance with previous findings [15,51,82,98] which demonstrated a higher level of social support was associated with better clinical outcomes. Consistent encouragement from a social support network could facilitate changes on an individual’s lifestyle [23], enhance patients’ quality of life and satisfaction from the provided care, improving treatment adherence, results in laboratory results (lower phosphate and potassium), and lead to better clinical outcomes [82,99]. As expected, patients with adequate dialysis and better adherence to phosphate binders possessed a lower serum phosphate level, emphasizing the importance of adequate dialysis and phosphate binders in optimizing control of serum phosphate.

Several limitations identified in this present study were the cross-sectional study design which limited the determination of causal relationships between the variables. We acknowledge that not all potential risk factors but rather only those which are routinely available were included in this study. Interviewer-administered questionnaires introduced bias and were highly dependent on the literacy and honesty of the subjects. We did not perform objective assessment on sleep such as polysomnography. Further studies with polysomnogram or other objective measures are needed. Despite the limitations present, this study highlighted several important findings that demand further in-depth research and investigations. 

## 5. Conclusions 

In conclusion, our findings reaffirmed poor compliance on serum phosphate levels among our hemodialysis patients. Besides, the present study also drew our attention to the role of diabetes mellitus in serum phosphate controls among HD patients, elucidating the need for healthcare professionals to monitor hyperphosphatemia closely among hemodialysis patients with diabetes mellitus. It is worth to note that 94% of the subjects in our study had an insufficient level of nutritional knowledge on phosphorus. Hence, healthcare professionals especially dietitians play an important role by providing interventions and increasing their awareness concerning this issue. Acknowledgement of barriers and factors affecting serum phosphate level compliance aids the provision of appropriate strategies and coping strategies that help to improve the clinical outcomes of HD patients. We hope by the identification of these non-conventional factors, namely social support, sleep duration and depression can assist the nephrology team in implementing a more comprehensive strategy in lessening hyperphosphatemia risk among the dialysis population.

## Figures and Tables

**Table 1:** Selected sociodemographic and clinical characteristics of patients (*n* = 217).

Characteristics		*n* (%)	Mean ± SD	Range
Age	<60 years old	121 (55.8)	57 ± 13	24–80
	≥60 years old	96 (44.2)		
Sex	Female	110 (50.2)		
	Male	108 (49.8)		
Marital status	Single	18 (8.3)		
	Married	170 (78.3)		
	Divorced	3 (1.4)		
	Widow/widower	26 (12.0)		
Educational level	No formal education	4 (1.8)		
	Primary	37 (17.1)		
	Secondary	104 (47.9)		
	Tertiary	72 (33.2)		
Employment status	Employed	51 (23.5)		
	Unemployed	64 (29.5)		
	Retired	102 (47.0)		
Monthly Household income (RM) *	B40 (<3860)	151 (69.6)		
	M40 (3860–8319)	46 (21.2)		
	T20 (≥8320)	20 (9.2)		
Duration of dialysis (months)			55.4 ± 48.7	3–360
Presence of Comorbidities ^†^	Hypertension	176 (81.1)		
	Diabetes mellitus	121 (55.8)		
Total BDI Score			5.76 ± 5.66	0.00–27.00
Level of Depression	None or minimal	165 (76.0)		
	Mild to moderate	44 (20.3)		
	Moderate to severe	8 (3.7)		
Level of Social Support **^‡^**	Family	190 (87.6)		
	Friends	127 (58.5)		
	Significant others	175 (80.6)		
PSQI score			6.15 ± 3.54	
Classification of Sleep Quality	Good sleepers	104 (46.6)		
	Poor sleepers	119 (53.4)		
Duration of Sleep (hour/day)	<5 hour/day	74 (34.1)	5.6 ± 1.8	
	5–6 hour/day	45 (20.7)		
	6–7 hour/day	46 (21.2)		
	>7 hour /day	52 (23.9)		
Creatinine (µmol/L)			950.6 ± 212.5	452.7–1021.5
Serum alkaline phosphatase (units/L)			385.0 ± 160.0	140–1120
Corrected serum calcium (mmol/L)	Low (<2.10)	36 (16.6)	2.23 ± 0.24	1.65–2.77
	Normal (2.10–2.37)	121 (55.8)		
	Elevated (>2.37)	60 (27.6)		
Intact parathyroid hormone (pg/mL)	<150	126 (58.1)	248.5 ± 163.2	118–452
	150–300	36 (16.6)		
	>300	55 (25.3)		
Dialysis Adequacy (Kt/V)	<1.2	25 (11.5)	1.42 ± 0.43	1.28–1.51
	>1.2	192 (88.5)		
Serum phosphate (mmol/L)	Normal (<1.6)	49 (22.6)	1.83 ± 0.50	0.78–3.37
	Elevated (≥1.6)	168 (77.4)		
Missing of phosphate binders	Yes	155 (71.4)		
	No	62 (28.6)		
Reasons of missing phosphate binders ^†^	Careless	138 (63.6)		
	Forgetful	112 (51.6)		
	High tablet burden	92 (42.4)		
	Gastrointestinal discomfort/Unpleasant taste	87 (40.1)		
Nutrition knowledge on phosphorus	<60%	204 (94.0)		
	≥60%	13 (6.0)		
Factors affecting optimal phosphate control ^†^ (patients’ perception)	Dialysis process alone	183 (84.3)		
	Low phosphorus diet alone	158 (72.8)		
	Phosphate binders alone	116 (53.5)		
	All the above	59 (27.2)		

Data were presented as Mean ± SD or *n* (%) * B40: bottom 40%; M40: middle 40%; T20: top 20%; Classified based on Eleventh Malaysia Plan (2016–2020), RM 1 was equivalent to approximately USD 0.25 at the time of data collection; ^†^ Multiple Responses; ^‡^ Patients who reported a high level of support in the respective subscales; PSQI: Pittsburgh Sleep Quality Index.

**Table 2:** Determinants of serum phosphate level among hemodialysis patients.

Variables	Simple Linear Regression		Multiple Linear Regression	
	Standardized Coefficients (β)	*p*	Standardized Coefficients (β)	*p*
Dialysis adequacy (Kt/V)	−0.455	0.007	−0.413	0.011
Adherence on Phosphate binders	−0.442	0.012	−0.368	0.021
Age	−0.334	0.015	−0.305	0.026
Sleep Duration	−0.378	0.032	−0.288	0.037
Presence of diabetes mellitus	0.326	0.035	0.272	0.038
Presence of depression	−0.358	0.033	−0.265	0.041
Social Support	−0.152	0.154	−0.141	0.044
Employment status	−0.271	0.037	-	-
Household income	0.259	0.041	-	-
Presence of hypertension	0.189	0.056	-	-
Duration of dialysis	0.176	0.124	-	
PSQI score	0.130	0.274	-	-
Nutritional knowledge on Phosphorus	−0.115	0.360	-	-

Variables with *p* < 0.25 in the simple linear regression model were included in the multiple linear regression analysis. Multiple linear regression model: R^2^ = 0.273, Adjusted R^2^ = 0.236, F (7, 217) = 12.4, *p* < 0.05.

**Table 3:** Serum phosphate of patients according to specific variables.

Variables	*n* (%)	Serum Phosphate (mmol/L) Mean ± SD	t or F Value ^†^
Dialysis adequacy (Kt/V)			
<1.2	25 (11.5)	2.18 ± 0.33	2.64 *
>1.2	192 (88.5)	1.78 ± 0.24	
Age (years)			
<60	121 (55.8)	1.93 ± 0.38	2.35 *
≥60	96 (44.2)	1.70 ± 0.61	
Presence of Diabetes			
Yes	121 (55.8)	1.89 ± 0.51	2.01 *
No	96 (44.2)	1.75 ± 0.54	
Level of Depression ^‡^			
None or minimal	165 (76.0)	1.90 ± 0.59 ^a^	5.02 *
Mild to moderate	44 (20.3)	1.59 ± 0.60 ^b^	
Moderate to severe	8 (3.7)	1.62 ± 0.45 ^b^	
Level of Social Support ^‡^			
Low or Moderate^#^	59 (27.2)	1.99 ± 0.61 ^a^	2.12 *
High	158 (72.8)	1.77 ± 0.64 ^b^	
Duration of Sleep (hour/day) ^‡^			
<5	74 (34.1)	2.04 ± 0.53 ^a^	5.68 *
5–6	45 (20.7)	1.86 ± 0.48 ^b^	
6–7	46 (21.2)	1.67 ± 0.48 ^c^	
>7	52 (23.9)	1.65 ± 0.51 ^c^	
Adhered to phosphate binders			
Yes	62 (28.6)	1.63 ± 0.46	2.48 *
No	155 (71.4)	1.91 ± 0.51	

^†^ Mean comparison between groups were computed using Student’s t test or one-way ANOVA whichever appropriate. * *p* < 0.05. **^#^** As there were only four patients with low perceived support, this was collapsed with the moderate perceived support. ^‡^ Different superscripts (^a, b, c^) indicate statistically different.
